# Predictors of psychosocial outcome of bipolar disorder: data from the Stanley Foundation Bipolar Network

**DOI:** 10.1186/s40345-019-0169-5

**Published:** 2019-12-16

**Authors:** Francis Bennett, Sophie Hodgetts, Andrew Close, Mark Frye, Heinz Grunze, Paul Keck, Ralph Kupka, Susan McElroy, Willem Nolen, Robert Post, Lars Schärer, Trisha Suppes, Aditya N. Sharma

**Affiliations:** 1 Institute of Neuroscience, Newcastle University, Newcastle upon Tyne, UK; 2 School of Psychology, University of Sunderland, Sunderland, UK; 3 Department of Psychiatry & Psychology, Mayo Clinic, Rochester, MN USA; 4 PMU Nuremberg & Psychiatrie Schwäbisch Hall, Schwäbisch Hall, Germany; 5 Linder Center of Hope, Mason, OH USA; 6 Biological Psychiatry Program, University of Cincinnati Medical College, Cincinnati, OH USA; 7 Department of Psychiatry, VU University Medical Center, Amsterdam, The Netherlands; 8 University of Groningen, University Medical Center Groningen, Groningen, The Netherlands; 9 Bipolar Collaborative Network, Bethesda, MD USA; 10 Department of Psychiatry, and Psychotherapy Medical Center, University of Freiburg, Faculty of Medicine, Freiburg im Breisgau, Germany; 11 School of Medicine and V.A. Palo Alto Health Care System Palo Alto, Stanford University, Palo Alto, CA USA; 12 Northumberland Tyne and Wear NHS Foundation Trust, Newcastle upon Tyne, UK; 13 Academic Psychiatry, Wolfson Research Centre, Campus for Ageing and Vitality, Newcastle University, Newcastle upon Tyne, NE4 5PL UK

**Keywords:** Bipolar disorder, Social functioning, Comorbidity

## Abstract

**Background:**

Impairments in psychosocial functioning have been demonstrated in 30–60% of adults with bipolar disorder (BD). However, the majority of studies investigating the effect of comorbid mental health disorders and age at onset outcomes in BD have focused on traditional outcome measures such as mood symptoms, mortality and treatment response. Therefore, this project aimed to investigate the impact of comorbid mental health disorders and age at onset on longitudinal psychosocial outcome in participants with BD.

**Method:**

Mixed effects modelling was conducted using data from the Stanley Foundation Bipolar Network. Baseline factors were entered into a model, with Global Assessment of Functioning (GAF) score as the longitudinal outcome measure. Relative model fits were calculated using Akaike’s Information Criterion.

**Results:**

No individual comorbidities predicted lower GAF scores, however an interaction effect was demonstrated between attention deficit hyperactivity disorder (ADHD) and any anxiety disorder (*t* = 2.180, *p* = 0.030). Participants with BD I vs BD II (*t* = 2.023, *p* = 0.044) and those in the lowest vs. highest income class (*t* = 2.266, *p* = 0.024) predicted lower GAF scores. Age at onset (*t* = 1.672, *p* = 0.095) did not significantly predict GAF scores.

**Conclusions:**

This is the first study to demonstrate the negative psychosocial effects of comorbid anxiety disorders and ADHD in BD. This study adds to the growing database suggesting that comorbid mental health disorders are a significant factor hindering psychosocial recovery.

## Background

Bipolar disorder (BD) is a mood disorder characterised by recurrent episodes of mania, hypomania, and depression, separated by periods of euthymia. Although not characterised by mood symptoms, psychosocial functioning appears to remain impaired during euthymia (Marangell et al. 2009). Psychosocial functioning is an essential component of a person’s quality of life (QoL), and includes social, psychological and occupational domains. In 2001, a landmark review found that between 30 and 60% of adults with BD had significant impairments in occupational and social functioning during periods of euthymia (MacQueen et al. 2001). A possible explanation for pervasive psychosocial dysfunction may be the illness itself or the high prevalence of comorbid mental disorders in BD (Post et al. 2015). However, research into BD has often overlooked the role of psychosocial functioning.

High rates of anxiety disorders (AnxD) comorbid with BD have been found consistently in both epidemiological (Merikangas et al. 2011) and clinical (Otto et al. 2006) samples. A recent review of 25 studies concluded that the lifetime risk of developing a comorbid AnxD was 46.8%, although epidemiological samples put this figure closer to 70% (Vazquez et al. 2014). Comorbid AnxD in subjects with BD are associated with a more severe illness course, including increased number of mood episodes, suicide attempts and hospitalizations, compared to BD alone (Merikangas et al. 2007). Despite this considerable evidence base, a recent review highlighted the current stagnation in AnxD comorbidity research, epitomised by the poor understanding of psychosocial factors, scarcity of treatment studies, and the lack of evidence base on antidepressants treating bipolar depression (Provencher et al. 2012). Although studies have shown associations between comorbid AnxD and poor psychosocial functioning (Scott et al. 2014), the picture is still far from clear.

Comorbid substance use disorders (SUD) are also prevalent the BD population, with epidemiological surveys putting lifetime prevalence of comorbid SUD at as high as 42.3% (Merikangas et al. 2007). However, with the exception of alcohol (Hobbs et al. 2011), the functional impacts and treatment options for individual SUD in BD have yet to be rigorously investigated.

Attention deficit hyperactivity disorder (ADHD) is a neurodevelopmental disorder, and its prevalence in BD varies widely with the age at which it is estimated. Approximately 20% of adult patients with ADHD also have bipolar disorder, while 10–20% of patients with bipolar disorder have adult ADHD (Brus et al. 2014). As with SUD and AnxD, ADHD comorbidity has been associated with increased number of mood episodes, higher rates of suicide attempts (McIntyre et al. 2010) and psychosocial dysfunction (Sentissi et al. 2008).

Mental health comorbidities in BD are more likely to be multiple than singular, with the World Mental Health Survey reporting a 62% lifetime prevalence of 3 or more comorbidities when strict Diagnostic and Statistical Manual of Mental Disorders-IV (DSM-IV) criteria were applied (Merikangas et al. 2011). However, research into the psychosocial effects of multiple comorbidities in BD is limited, often due to lack of power in subgroup analyses (Sentissi et al. 2008). Although there is clear evidence of increased rates of both individual and multiple comorbidities in BD, their impacts on day-to-day functioning of people with BD have yet to be established.

Earlier age at onset (AAO) of BD is consistently linked with poorer clinical outcomes, including rapid cycling, greater number of mood episodes and increased risk of suicide (Leverich et al. 2007). Some studies have linked psychosocial dysfunction with younger (< 18 years) AAO (Perlis et al. 2009), although this finding is not consistent (Martinez-Aran et al. 2007). The studies that have focused on younger AAO suggest that psychosocial impairment is due to earlier disruption in the development of interpersonal skills needed to build and maintain healthy relationships as patients grow older (Levy and Manove 2012). However, while younger AAO is associated with an adverse course of illness in adulthood (Leverich et al. 2007), how these may be related to psychosocial functioning has received little attention (Perlis et al. 2009).

In this post hoc analysis we used data from an established database (Stanley Foundation Bipolar Network) (Post et al. 2001) to estimate the effects of comorbid mental health disorders and AAO on psychosocial outcomes in participants with BD. A novel aspect to this study is the use of statistical modelling techniques, specifically mixed effects modelling (MEM). Modelling has been used extensively in the behavioural sciences (Gerhard et al. 1995), while recognition of its utility in medical fields is growing. The STEP-BD group used MEM in two papers assessing psychosocial outcome measures (Otto et al. 2006; Perlis et al. 2009). Complex data sets, such as the SFBN, often violate the assumptions of general linear modelling; in particular, statistical assumptions relating to independence between observations are violated. In contrast, mixed effects modelling is particularly suited to data sets involving measurements obtained from individual patients that share socioeconomic, demographic or biomedical characteristics. Therefore, a key advantage of MEM is that it calculates how much of the variance in the sample is explained by each factor and, crucially, what residual variance is left. Most clinical studies lack sufficient numbers to run the more complex models, but the longitudinal nature and size of the SFBN cohort makes this a unique opportunity in psychiatric research.

### Aims of the study

Psychosocial functioning is key to understanding the overall impact mental health disorders can have on an individual. In BD research, comorbid mental health disorders and age at onset have both been extensively investigated with traditional outcome measures such as mood symptoms, mortality and treatment response. In this study, we used Mixed-Effects Modelling on data collected by the Stanley Foundation Bipolar Network. The study aimed to demonstrate the effect of comorbid mental health disorders and age of onset on psychosocial functioning in participants with BD.

## Methods

### Baseline assessments

Information on comorbid mental health disorders and AAO was collected utilizing items from Structured Clinical Interview for DSM-IV-Patient (SCID-P) and Patient Questionnaire (PQ). AAO was defined as age at first hypomanic, manic, or depressive symptoms that were associated with functional impairment. AAO for BD was collected from the SCID-P, which a previous SFBN paper showed to be highly correlated with self-report from PQ (*r* = 0.80) (Leverich et al. 2007).

### Longitudinal outcome

The Global Assessment of Functioning (GAF) is a well-validated instrument, which constitutes Axis V of DSM-IV. It has been used extensively in BD research and has good inter-rater reliability (Jones et al. 1995). A criticism of the GAF is that it confounds mood and functioning by including mood symptoms in the scale descriptions. In the SFBN this was addressed by ensuring clinicians only used the GAF to rate functioning. Following an interview, clinicians rated the participant’s global functioning on a scale of 0–100. Three scores were recorded: best, worst (since last visit) and current. This study only included current score in order not to introduce a selection bias.

### Data selection

**Fig. 1:**
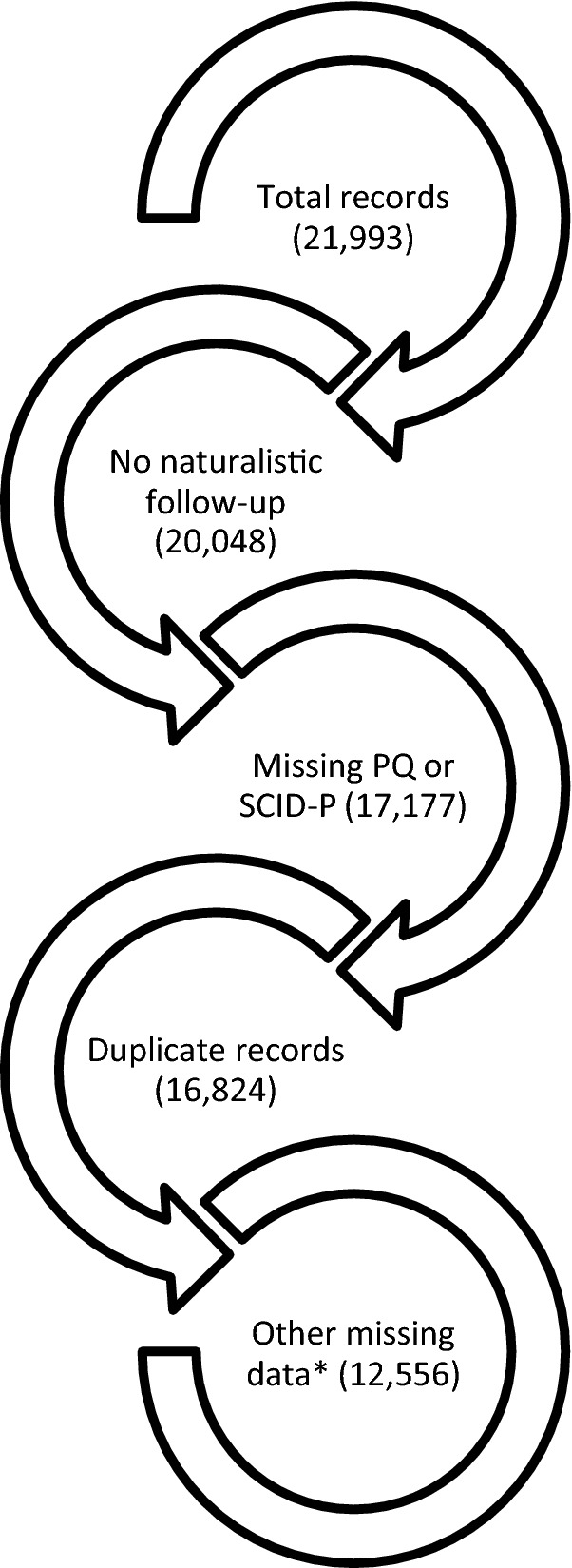
Flow-chart of exclusion criteria (GAF record number) *No GAF score/inadequate information, no gender, no income, no education, no age at first treatment for mania, no BD type, no AAO, negative duration of untreated bipolar

The total sample study size in the original data was n = 648, with 21,993 GAF records. Only participants with complete data were included (see Fig. 1) which left a final sample size of n = 469, with 12,556 GAF records.

### Data analysis

Mixed-effects models (MEMs) were developed using the strategy described by Pinheiro et al. (2017) to examine variation in psychosocial dysfunction among participants at follow-up. A GAF score for each participant was obtained at baseline and at follow-up. We implemented the methodology proposed by Vickers and Altman (2001) and used GAF at baseline in order to control for differences in the mean psychosocial function between sites in Europe (Netherlands and Germany) and USA. The impact of BD subtype of each participant, AAO and the mental health comorbidities on psychosocial outcome were also determined. The influences of demographic characteristics of participants were assessed by including sex and income as independent explanatory variables. Independent explanatory variables were assessed within models additively. The potential impact of multiple interactions between AnxD, SUD and ADHD on psychosocial outcome was also explored. Individuals were grouped according to their respective locations (Europe or USA). Study location was then specified as a random-effect within the model in order to capture potential sources of unmeasured variation in psychosocial outcome. In addition, GAF at baseline within the random-effects structure was incorporated to generate a random intercept and random slope model that controlled for variation in overall mean and gradient between locations. Multi-model inference (Burnham and Anderson 2002) was used to compare models with additive terms to those models that incorporated both additive and interaction actions terms. A model with the lowest Akaike’s Information Criterion (AIC) (Akaike 1973) was considered to be the model that best described variation within data.

## Results

### Preliminary analysis

There were greater numbers of participants included in the final analysis from USA (n = 332) than from Europe (n = 137). However, a Chi-squared test of independence did not find differences in the distribution of genders between USA (female:male, 189:124) and Europe (female:male, 70:63) (*Χ*_2_ = 2.17, *p* = 0.141). In contrast, the distribution of mental health comorbidities, namely AnxD (*Χ*_2_ = 22.98, *p* = < 0.001), ADHD (*Χ*_2_ = 8.23, *p* = 0.004) and SUD (*Χ*_2_ = 20.29, *p* < 0.001) all differed significantly between locations, and were higher in the US than in Europe. No significant differences in the distribution of BD subtypes (*Χ*_2_ = 0.44, *p* = 0.081), or income classes (*Χ*_2_ = 0.58, *p* = 0.748) was observed between USA and Europe. Demographic information for the whole sample is given in Table 1, while clinical and GAF summaries are given in Table 2.

**Table 1:** Demographic characteristics and comorbidity in USA vs Europe

	USA (n = 332, 70.8%)	Europe (n = 137, 29.2%)	Total (n = 469, 100%)	*χ*^2^	*p*-value
Gender					
Female	59.9	52.6	57.8		
Male	40.1	47.4	42.2	2.17	0.141
Income ($)					
< 20,000	38.9	36.5	38.2		
20,000–59,999	38.6	42.3	39.7		
> 60,000	22.6	21.2	22.2	0.58	0.748
BD type					
BD I	84.3	83.2	84.0		
BD II	13.6	15.3	14.1		
BD cyclothymic	2.1	1.5	1.9	0.44	0.081
Comorbidity					
AnxD	52.7	28.5	45.6	22.98	< 0.001
*Panic disorder*	35.8	19.7	31.1		
*Chronic AnxD*	33.4	16.8	28.6		
*OCD*	22.9	4.4	17.5		
ADHD	16.6	6.6	13.6	8.23	0.004
SUD	47.3	24.8	40.5	20.29	< 0.001
AnxD + SUD	26.2	9.5	19.2		
ADHD + SUD	9.9	2.9	7.9		
ADHD + AnxD	13.3	5.8	11.1

**Table 2:** Sample demographics, mean GAF at baseline, and follow-up by location, sex and BD subtype

Location	Sex	DSM-IV bipolar disorder subtype	Mean follow-up attendance (%)	Mean GAF baseline	Mean GAF follow-up	Sample size (%)
Europe	Female	BD I	30.53	63.75	66.68	57 (12.5)
Europe	Female	BD II	40.85	73.08	71.62	13 (2.9)
Europe	Female	BD NOS	41.00	70.00	32.50	2 (0.40)
Europe	Male	BD I	31.04	64.16	71.02	56 (12.3)
Europe	Male	BD II	38.86	67.57	80	7 (1.5)
Europe	Male	BD NOS	NA	NA	NA	NA
USA	Female	BD I	24.22	62.84	65.25	165 (36.3)
USA	Female	BD II	24.08	64.33	68.46	24 (5.3)
USA	Female	BD NOS	11.75	54.00	62.75	4 (0.9)
USA	Male	BD I	28.47	61.02	65.03	104 (22.9)
USA	Male	BD II	26.50	63.20	67.70	20 (4.4)
USA	Male	BD NOS	42.33	71.00	61.00	3 (0.7)

Preliminary analysis undertaken using a Wilcoxon Signed-Rank Test indicated that the location of mean of GAF measured at baseline was significantly different between locations (W = 23,472, *p *= 0.0325), and higher in Europe. The location of the mean GAF derived at follow-up was also found to be significantly different between locations (W = 24,844, *p *= 0.0012). It was not possible to explore the impact of educational attainment in the assessment of variation in GAF due to imbalance in sample size between the lower and higher levels of attainment. Similarly, participants with BD NOS were removed from further analyses due to insufficient sample size (n = 9). Subsequently, variation in psychosocial function among 446 participants was analysed.

### Statistical analysis of global assessment of functioning (GAF)

The relative fits of MEMs were compared using multi-model inference. A model was considered a plausible fit to data if the difference in AIC between models did not exceed ∆_I_ < 2 (Table 3). The model incorporating an interaction between AnxD and ADHD was considered to be the best candidate model from the specific set of models compared (AIC = 3582.256, ∆_I_ = 0.000) (Table 3). The model incorporating comorbidities additively was ranked second within the model set (AIC = 3588.113, ∆_I_ = 5.361) and models that examined interactions between ADHD and SUD (AIC = 3593.008, ∆_I_ = 10.256) and Anxiety Disorders and SUD (AIC = 3594.422, ∆_I_ = 11.670) were ranked third and fourth, respectively.

**Table 3:** Multi-model inference indicating that the model incorporating two interactions terms best describes the data

Fixed effects structure (comorbidities)	*df*	Log likelihood	AIC	∆_i_	wt
Anxiety disorders and ADHD	24	− 1762.376	3582.752	0.000	0.928
Additive terms	25	− 1769.056	3588.113	5.361	0.064
ADHD and substance use disorder	23	− 1773.504	3593.008	10.256	0.006
Anxiety disorder and substance use disorder	27	− 1770.211	3594.422	11.670	0.003

Results generated by MEMs examining variation in GAF scores at follow-up, and incorporating an interaction between AnxD and ADHD are presented in Table 4. The global mean (Intercept) GAF at follow-up for participants with BDI (reference level) was 67.757 and was significantly different from zero (*t* = 25.456, *p* ≤ 0.000). Participants with BDII were found to have significantly higher GAF scores at follow-up in comparison to participants with BDI (*t* = 2.023, *p *= 0.044). Baseline GAF for participants was found to be significantly and positively associated with GAF at follow-up (*t *= 3.586, = *p* ≤ 0.000). In addition, a significant negative association was found for participants with BDII and psychosocial function at baseline (*t* = − 2.059180, *p* = 0.017), suggesting lower psychosocial functioning in BDII patients at baseline. In contrast, there was no significant relationship between BD subtype and AAO and psychosocial function at follow-up. Individuals grouped within the highest income class were significantly associated with higher GAF at follow-up in comparison to participants in the lowest class (*t* = 2.266, *p* = 0.024). In contrast, there was no significant difference at follow-up between participants within income class-1 and income class-2. Comorbidities included as additive terms within the model, AnxD, SUD and ADHD, did not significantly affect psychosocial function at follow-up. However, when AnxD and ADHD were incorporated as an interaction term, a significant negative effect on GAF at follow-up was observed (*t *= 2.180, *p* = 0.030). The mean GAF at follow-up was not significantly affected by variation in duration of follow-up, and no significant differences were observed in GAF score at follow-up between females and males.

**Table 4:** Mean GAF score at follow-up (intercept) and change with respect to explanatory variables

Parameter	Estimate	95% CI	*se*	*df*	*t*-value	*p*-value
Intercept	67.757	62.225, 73.518	2.662	431	25.456	≤ 0.000
BD: subtype-2	3.191	0.146, 0.645	1.578	431	2.023	0.044
Clinical follow-up attendance	− 0.008	0.407, 6.908	0.029	431	− 0.287	0.774
GAF baseline	0.410	0.019, 0.308	0.114	431	3.586	≤ 0.000
Age of onset	0.120	− 4.657, 2.122	0.072	431	1.672	0.095
Anxiety disorder	− 2.162	− 4.823, 0.498	1.354	431	− 1.597	0.111
ADHD	1.279	− 2.979, 5.537	2.166	431	0.591	0.555
Substance use disorder	1.552	− 0.869, 3.973	1.232	431	1.260	0.208
Sex: male	0.771	− 1.650, 3.192	1.232	431	0.626	0.532
Income-2^a^	− 0.551	− 3.261, 2.160	1.379	431	− 0.399	0.690
Income-3^a^	3.572	0.474, 6.670	1.576	431	2.266	0.024
BD subtype-2: GAF baseline	− 0.265	− 0.518, − 0.012	0.129	431	− 2.059	0.040
BD subtype-2: age of onset	− 0.244	− 0.518, − 0.029	0.139	431	− 1.758	0.080
Anxiety disorder: ADHD	− 6.953	− 13.222,− 0.683	3.190	431	− 2.180	0.030

^a^Income-2: 20,000–59,999 (USD), income-3: > 60,000 (USD)

## Discussion

The aim of this study was to investigate the impact of comorbid mental disorders and AAO on psychosocial functioning in an international sample of participants with BD. These factors were entered into MEM with GAF score as the longitudinal psychosocial outcome measure. The principle finding is that BD comorbid with ADHD and AnxD predicted lower GAF scores, particularly in BDI. Due to the influence of confounding collinearity between explanatory variables, it was not possible to construct a single model that examined the multiplicative effects between all comorbidities simultaneously. Nevertheless, a set of MEMs were developed that first examined the effects of comorbidities additively, in addition to models that examined the multiplicative effects between comorbidities independently. Individual comorbidities were explored independently within the final model, with no single comorbidity reaching statistical significance in predicting psychosocial outcome.

The high rate of comorbid AnxD in BD (45.6%) demonstrated in this study is a well-replicated finding (Simon et al. 2004). BD and comorbid AnxD alone did not predict poorer psychosocial function in the present study. This adds to a limited evidence base, and warrants further investigation. One explanation for this finding could be that the diagnostic criteria used did not capture the full spectrum of anxiety states and their effects.

As discussed earlier, rates of comorbid ADHD and BD vary widely according to age group. In the current study comorbid ADHD (13.6%) was less prevalent than both AnxD (45.6%) and SUD (40.5%). Currently, only two previous papers have looked at psychosocial outcome in comorbid ADHD and BD (Sentissi et al. 2008; Wilens et al. 2003). A study using more specific social functioning measures (SF-36 Health Survey and Social Adjustment Scale) found that poorer scores were predicted by ADHD comorbidity (Sentissi et al. 2008). Another smaller study found only a trend towards poorer global psychosocial functioning (GAF) in the ADHD group (Wilens et al. 2003). Alongside the current results, this may suggest a specific social dysfunction related to ADHD comorbidity, since both studies also found that attention domains were significantly more impaired than hyperactivity domains in the comorbid ADHD group (Sentissi et al. 2008; Wilens et al. 2003). The evidence regarding specific attention deficits and social dysfunction needs further replication, however, it may be that the relationship between ADHD and BD depends upon to specific aspects of attention that are affected.

The current study did not find a predictive effect of comorbid SUD on psychosocial dysfunction in BD. It may have been an over-simplification to analyse all types of SUD together in this study. Alcohol and cannabis were the most widely abused substances in the SFBN sample, but a recent review highlighted the differential impacts of these two SUDs in BD (Cerullo and Strakowski 2007). The current results are especially interesting when looking at the new DSM-5 (American Psychiatric Association 2013) criteria for SUD, which no longer differentiate between substance abuse and dependence. Instead, SUD severity is classified by the number of criteria met. Of the 11 criteria listed three relate to social impairment, making it possible to have a SUD diagnosis without exhibiting any social dysfunction.

To the authors’ knowledge, this is the first paper that examines the effect of comorbid ADHD and AnxD on psychosocial outcome in BD. A key similarity between comorbid ADHD and AnxD individually with BD is the persistence of their symptoms during periods of euthymia (Bernardi et al. 2010). This is likely to hinder psychosocial recovery between mood episodes, leading to reduced quality of life. The chronic nature of comorbid ADHD can lead to underreporting the functional impact of symptoms, (Sandra Kooij et al. 2008) although this has yet to be demonstrated in comorbid AnxD. We found that participants within the SFBN cohort with AnxD were significantly more likely to also have ADHD (OR = 5.821, 95% CI 3.00–12.233). This suggests a heavy burden of ADHD in those participants with comorbid AnxD and BD. While the detrimental influence of such a potent combination of mental health disorders is perhaps not surprising, the underlying pathophysiological mechanisms merit further investigation.

The implications for treatment also merit discussion. There is very sparse guidance on treatment of comorbid mental health disorders in BD. The UK National Institute for Health and Care Excellence (NICE) guidelines merely state that clinicians should be “alert to the potential for drug interactions and use clinical judgement” (National Collaborating Centre for Mental Health 2014). American Psychiatric Association guidelines state treatment for comorbid AnxD and BD should “proceed concurrently” (American Psychiatric Association 2002). Canadian/ISBD guidelines state that when ADHD is comorbid with mood disorders then the condition causing most impairment should be treated first (Bond et al. 2012). This scenario can pose challenges for clinicians, as all three conditions are likely to be interacting to worsen the course of the other. In comorbid BD and ADHD, there is evidence in both children and adults to suggest a mood stabiliser and stimulant are effective in treating their respective symptoms (Findling et al. 2007). However, in adults there is also evidence of stimulant associated (hypo)mania (Wingo and Ghaemi 2008). On the other hand, one could also consider psychological treatment for the comorbid ADHD such as CBT or skills’ training, which is not likely to harm treatment of BD. The presence of an additional AnxD with ADHD and BD further complicates matters, as dual-action monoamine reuptake inhibitors also risk precipitating a (hypo)manic episode, although again this would not be the case for psychological treatments such as CBT (Goodwin et al. 2016).

Participants with BDII were significantly more likely on average to exhibit higher GAF scores at follow-up than participants with BDI. This is in line with previous findings showing higher GAF scores in BDII patients compared to BDI patients, despite similar illness duration, family history, previous suicide attempts, and overall psychiatric comorbidity (Dell’Osso et al. 2017b). This also indirectly supports the notion that psychotic symptoms that are particularly prevalent in BDI patients, may contribute to the lower GAF scores seen in this group (Dell’Osso et al. 2017a). This finding is also in line with the core criteria for mania as set out in DSM 5, that the mood episode impairs social or occupational functioning. AAO did not significantly influence the mean GAF at follow-up. As discussed previously, there is still sparse literature on the effects of earlier AAO on psychosocial functioning and their conclusions are varied (Martinez-Aran et al. 2007; Perlis et al. 2009). It is important to note that this study used age at first (hypo)manic symptoms or depressive symptoms with dysfunction to define AAO.

Participants in the highest income class were significantly more likely to yield better outcomes than those in the lowest income class. Numerous studies have shown that low socioeconomic status (SES) has significant detrimental impacts on mental health at a population level (Lorant et al. 2003). However, there is little evidence regarding the effects of SES on psychosocial functioning in BD. One study found no association between education levels and functional outcome (Schoeyen et al. 2011), although the outcome was ‘disability pension’; a fairly crude measure of psychosocial functioning.

The confounding effect observed between explanatory variables may be an important reason as to why individual assessment of the effects of comorbidities appeared to be spurious. Nevertheless, when we assess the individual effects in conjunction with the interaction term we are able to ascertain the overall effect of mental health comorbidities on psychosocial outcomes. As our results indicate, those with multiple comorbidities (AnxD and ADHD) experience significantly reduced psychosocial function at follow-up.

The present study is not without limitation. First, while it is important that researchers use standardised scoring instruments in order to reduce bias and increase the replicability of their findings, the GAF has several limitations. Indeed, it has been suggested that it lacks the sensitivity to assess longitudinal change in an individual patient (Soderberg et al. 2005). Moreover, the GAF is global measure, and as such fails to capture the contributions of each specific dimension of psychosocial functioning to overall dysfunction. The GAF also specifically excludes functional impairment due to “physical or environmental limitations”, yet it is difficult to see how raters can distinguish aetiologically between physical or environmental and psychosocial factors.

A second critical limitation to this study is that it did not control for mood state. The possible impact of mood state on the present findings is two-fold. Firstly, depressive symptoms, even more than manic, are strong predictors of poor psychosocial functioning (Morriss et al. 2013). However, in the present study, it is not possible to replicate this relationship. Secondly, it has been shown that the incidence rates of comorbidities are sensitive to mood state, when assessed cross-sectionally (Post et al. 2018) as in the present study. It is also important to note that this study did not include medical comorbidities. These certainly play a significant role in psychosocial dysfunction but were beyond the scope of the current paper.

A third critical limitation is that the comorbid conditions were characterised by the presence of a lifetime diagnosis, and whether patients continued to manifest these difficulties consistently over their lifetime is not known. This limitation would be of particular importance for ADHD, as a substantial group of patients may have the comorbidity in childhood and/or adolescence, but no longer in adulthood. Similarly, severity of each of the comorbid problems was not assessed. Any anxiety-specific disorder was considered for inclusion in the category, and how individual anxiety disorders or their combination may have affected the relationship to function cannot be determined in this study. In addition, while the incidence of comorbidities in this study population where similar to those reported in the literature, the fact that the population was drawn largely from academic tertiary care facilities may limit the generalizability of the results and conclusions to other populations.

It is also important to challenge some of the assumptions that may be drawn from this paper, including the idea of a unidirectional relationship between the significant predictors and psychosocial dysfunction. As this study was conducted in adults, with data on predictors collected retrospectively, the relationships inferred are not conclusive. Future work should aim to clarify these findings using prospective studies starting in childhood to determine differences between those who do and do not go on to develop BD. This will help to inform the development of interventions aimed specifically at psychosocial dysfunction.

## Conclusion

In summary, this project has demonstrated the negative impact of comorbid AnxD and ADHD on psychosocial functioning in BD. Both inpatient and population studies have demonstrated the alarmingly high prevalence of comorbid disorders in BD, however current guidelines offer little help to clinicians when managing these disorders. Our findings suggest a unique interaction between AnxD and ADHD in BD, which will need further research to both replicate and explore this relationship and ascertain optimal therapeutic interventions. Resolution of clinical symptoms in BD is rarely followed by recovery of psychosocial functioning, and this study has added insights into the role of AnxD and ADHD comorbid disorders in impairing global psychosocial functioning.

## Data Availability

The datasets used and/or analysed during the current study are available from the corresponding author on reasonable request.
